# Changes in Motor-Related Cortical Activity Following Deep Brain Stimulation for Parkinson’s Disease Detected by Functional Near Infrared Spectroscopy: A Pilot Study

**DOI:** 10.3389/fnhum.2016.00629

**Published:** 2016-12-12

**Authors:** Takashi Morishita, Masa-aki Higuchi, Kazuya Saita, Yoshio Tsuboi, Hiroshi Abe, Tooru Inoue

**Affiliations:** ^1^Department of Neurosurgery, Faculty of Medicine, Fukuoka UniversityFukuoka, Japan; ^2^Department of Neurology, Faculty of Medicine, Fukuoka UniversityFukuoka, Japan; ^3^Department of Rehabilitation, Faculty of Medicine, Fukuoka UniversityFukuoka, Japan

**Keywords:** Parkinson’s disease, near infrared spectroscopy, deep brain stimulation, neuronal circuit, neuroplasticity

## Abstract

It remains unclear how deep brain stimulation (DBS) modulates the global neuronal network involving cortical activity. We aimed to evaluate changes in cortical activity in six (two men; four women) patients with Parkinson’s disease (PD) who underwent unilateral globus pallidus interna (GPI) DBS surgery using a multi-channel near infrared spectroscopy (NIRS) system. As five of the patients were right-handed, DBS was performed on the left in these five cases. The mean age was 66.8 ± 4.0 years. The unified Parkinson’s disease rating scale (UPDRS) motor scores were evaluated at baseline and 1- and 6-month follow-up. Task-related NIRS experiments applying the block design were performed at baseline and 1-month follow-up. The mean of the total UPDRS motor score was 48.5 ± 11.1 in the off-medication state preoperatively. Postoperatively, total UPDRS motor scores improved to 26.8 ± 16.6 (*p* < 0.05) and 22.2 ± 8.6 (*p* < 0.05) at 1- and 6-month follow-up, respectively. A task-related NIRS experiment showed a postoperative increase in the cortical activity of the prefrontal cortex comparable to the preoperative state. To our knowledge, this is the first study to use a multi-channel NIRS system for PD patients treated with DBS. In this pilot study, we showed changes in motor-associated cortical activities following DBS surgery. Therapeutic DBS was concluded to have promoted the underlying neuronal network remodeling.

## Introduction

There have been numerous neuroimaging studies concerning the pathophysiology of Parkinson’s disease (PD; Weingarten et al., 2015; Al-Radaideh and Rababah, 2016), and various aspects of PD came to be investigated with the development of neuroimaging technologies, especially magnetic resonance imaging (MRI). These MRI approaches include task-related functional MRI (fMRI), resting-state fMRI, and diffusion tensor imaging. In addition to these neuroimaging technologies, deep brain stimulation (DBS) surgery has been used to investigate the pathophysiology of PD. Although the literature includes a variety of MRI studies of patients with PD, due to metal artifacts and safety issues, MRI should be performed in patients who have undergone DBS surgery with caution. Newer generation DBS systems are supposedly compatible with MRI; however, disastrous complications have been reported (Henderson et al., 2005; Zrinzo et al., 2011).

Near-infrared spectroscopy (NIRS) has been increasingly being applied in studies of stroke, epilepsy and neurorehabilitation (Obrig, 2014). The NIRS system measures the oxygenation levels of the target area by detecting the characteristic absorption spectra of hemoglobin through near-infrared light probes, and this hemoglobin response has been considered to reflect the neuronal activity in the cerebral cortex based on the neurovascular coupling mechanism. In general, neuronal activation increases the oxy-hemoglobin (HbO) and decreases deoxy-hemoglobin (HHb) levels (Devor et al., 2012). The NIRS system cannot measure activity in deep brain areas such as the basal ganglia; however, there are advantages of functional near infrared spectroscopy (fNIRS) over fMRI, such as the portability and safety of the system. Recent studies using multi-channel NIRS have revealed hemodynamic responses to various motor tasks such as the postural control task (Mihara et al., 2012) and pursuit rotor task (Hatakenaka et al., 2007). These studies reveal the changes in the cortical activities following rehabilitation and/or motor skill learning, and multi-channel NIRS has the potential to measure cortical activity changes following intervention. Of particular relevance to the present study, it should be noted that the multi-channel NIRS system enables the safe measurement of cortical activity in DBS patients.

Several studies have suggested that DBS modulates the global neuronal network of cortical activity, and recent studies show that DBS may disconnect the abnormal coupling of the oscillation activities between the basal ganglia and the motor cortex (de Hemptinne et al., 2015). We hypothesized that the abnormal activity in the neuronal circuits ultimately affects the neurovascular reactivity in the cerebral cortex of PD patients. Therefore, therapeutic DBS may address the pathologically altered cortical activity (neurovascular reactivity) especially in the primary motor cortex, and NIRS may safely detect changes in the cortical activity between pre- and post-DBS therapy. In this pilot study, we aimed to evaluate changes in cortical activity following DBS surgery using a multi-channel NIRS system.

## Materials and Methods

### Study Design

Data were collected from six consecutive patients with PD (two men; four women), recruited between April and November 2015, who underwent DBS surgery at our institution. This study was carried out in accordance with the permission from our institutional review board (IRB) of Fukuoka University Hospital, and written informed consent was obtained from all study participants. DBS candidates were first admitted to the Department of Neurology for detailed assessment. Preoperatively, we performed cognitive and neuropsychological evaluations, and patients with cognitive or psychological issues were excluded from the study.

The mean age of our cohort was 66.8 ± 4.0 years, and the mean disease duration was 12.2 ± 7.6 years at baseline. DBS surgery was performed on the left side for five right-handed patients. The mean number of preoperative levodopa equivalent doses (LEDs) was 1017.3 ± 306.0. One patient underwent right-sided DBS despite right-handedness (case 5), and another patient underwent left-sided DBS despite left-handedness (case 6). All patients were cognitively intact, as the mean Mini Mental State Examination (MMSE) score was 28.0 ± 2.1. These demographic data are summarized in Table 1.

**Table 1:** **Demographic data**.

Case	Age (years)	Duration (years)	Sex	Handedness	DBS side	MMSE	LED
1	67	9	Female	Right	Left	30	1158
2	74	25	Male	Right	Left	30	870
3	65	8	Female	Right	Left	28	700
4	67	7	Female	Right	Left	26	825
5	62	6	Male	Right	Right	29	998
6	66	18	Female	Left	Left	25	1555
**Mean ± SD**	66.8 ± 4.0	12.2 ± 7.6				28.0 ± 2.1	1017.3 ± 306.0

*MMSE, Mini Mental State Examination; LED, levodopa equivalent dose; SD, standard deviation*.

Unified Parkinson’s Disease Rating Scale (UPDRS) motor scale (Part III) scores were evaluated in the off- and on-medication conditions to predict the clinical efficacy of DBS surgery. UPDRS motor assessment in the off-medication condition was performed following at least 12 h of levodopa and dopamine agonist washout, and patients took carbidopa/levodopa (25 mg/100 mg) for evaluation in the on-medication state. Each case was discussed at the interdisciplinary team meeting for DBS candidacy. In this study, all patients underwent unilateral DBS implantation in the globus pallidus interna (GPI), and the DBS side was selected based on multiple factors, such as handedness and patient-specific symptoms.

In patients with PD who underwent DBS surgery, UPDRS Part III scores were evaluated with on-DBS conditions in the off-medication state 1 and 6 months postoperatively, and all adverse events were prospectively recorded. The UPDRS Part III scores were compared between pre- and postoperative states using the Wilcoxon signed rank test. An NIRS experiment was performed pre- and 1-month-post-DBS surgery. The details of this NIRS experiment are described below.

### Surgical Procedure

A high-resolution volumetric MRI study was performed for stereotactic planning, and the obtained MRI sequences included T1-weighted images with contrast and fast gray matter acquisition T1 inversion recovery (Sudhyadhom et al., 2009). Stereotactic planning was performed 1 day prior to DBS implantation using commercial software (iPlan Stereotaxy, BrainLab, Germany). After anchoring the Cartesian coordinate system determined by the anterior commissure, the posterior commissure, and the midline plane, we planned the trajectory of microelectrodes such that they passed through the ventrolateral area of the GPI, terminated in the optic tract, and avoided the blood vessels, lateral ventricle and sulci.

All antiparkinsonian medications were stopped so that patients were in the off-medication state during the procedure. On the morning of surgery, a Leksell frame was applied, and the patient was transferred to a computed tomography scan site for stereotactic imaging. The computed tomography images were merged with the MRI images, and the preoperative DBS planning was finalized.

Patients were awake during the intracranial DBS lead implantation. In the operating room, the head was fixed to the operating table. A 5.5 cm linear skin incision was made, and a burr-hole was fashioned in the skull. Once the dura was opened, a microelectrode recording procedure was performed to map out the GPI, and then a DBS lead (Model 3387, Medtronic Inc., USA) was implanted for the macrostimulation procedure. A macrostimulation procedure was performed to confirm the clinical effects and threshold levels of stimulation-induced side effects. An implantable pulse generator (Activa SC, Medtronic Inc., Fridley, MN, USA) was implanted on the same day following the intracranial lead implantation. DBS programming was meticulously performed for a few weeks in a hospital setting, and continued monthly for 6 months at the neurology clinic.

### Functional Imaging Experiment

**Figure 1:**
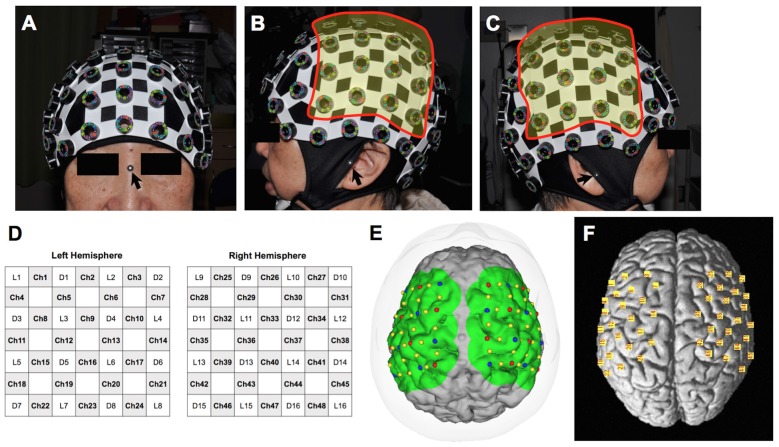
**Probe position on the head and estimated channel position on the individual and model brains. (A)** Anterior view of the head with the cap. The arrow indicates the nasion where a landmark is for spatial registration. **(B)** Lateral view from the left. Probes were attached to 16 holes in the highlighted area. The arrow indicates the left tragus. **(C)** Lateral view from the right. Probes were attached to 16 holes in the highlighted area. The arrow indicates the right tragus. **(D)** Schema of the probe positioning. L, light source; D, detector; Ch, channel. **(E)** Estimated channel position on the 3-D model of the patient’s brain created from a volumetric T1-weighted image. Red and blue dots indicate the light sources and detectors, respectively. **(F)** Estimated channel position (yellow squares) on the model brain based on the Montreal Neurological Institute coordinate system.

We used a continuous-wave NIRS system (FOIRE-3000, Shimadzu, Japan) for functional imaging. The wavelengths of the near-infrared light were 780, 805 and 830 nm, and the sampling rate was set at 7.8 Hz (time resolution: 130 ms). We attached 32 optodes (16 light sources and 16 detectors) to a head cap bilaterally over the frontal and parietal areas, forming a total of 48 channels (24 channels for each hemisphere). The interoptode distance and the inter-channel distance were 3.0 cm and 2.1 cm, respectively. Regions of interest primarily included the primary motor cortex. Prior to starting the experiment, we took pictures of the patient’s head with the head cap and the three-dimensional digitizer from 15 perspectives using a high-resolution digital camera for spatial registration (Figure 1).

A block design was applied for data acquisition. While in a sitting position, each patient was instructed to open and close the hand contralateral to the operative side for 15 s at comfortable speed, and rest for 30 s between this motor task for each cycle of 45 s. This motor task was repeated seven times in each session. The NIRS system acquired values of HbO and HHb levels following changes in its cortical concentration based on the modified Beer-Lambert law.

**Figure 2:**
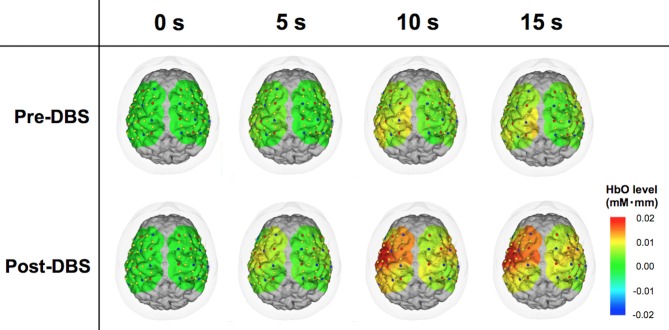
**Comparison between pre- and post-deep brain stimulation (DBS) cortical activity in a representative case.** The color map representing cortical activity superimposed onto the three-dimensional brain model was produced from the patient’s T1-weighted magnetic resonance imaging (MRI) image. Red and blue dots indicate the light sources and detectors, respectively. Yellow dots are channels formed by the combinations of the light sources and receivers. These images demonstrate the dynamic oxy-hemoglobin (HbO) response to motor task at 0, 5, 10 and 15 s. At 0 s, the levels of the measured areas were all corrected to zero. Cortical activity in the wide areas of operated-upon hemisphere increased following DBS surgery.

**Figure 3:**
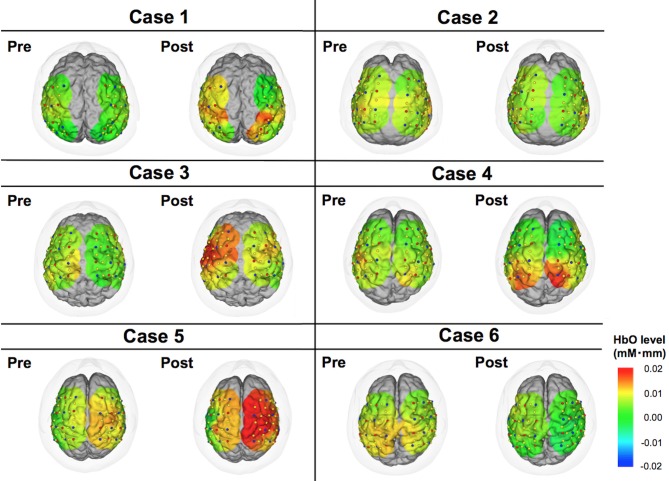
**Color maps of HbO levels comparing pre- vs. post-DBS states.** These images indicate cortical activities at 15 s from the start of motor task. Relative changes compared to the status at 0 s are demonstrated in this figure.

**Figure 4:**
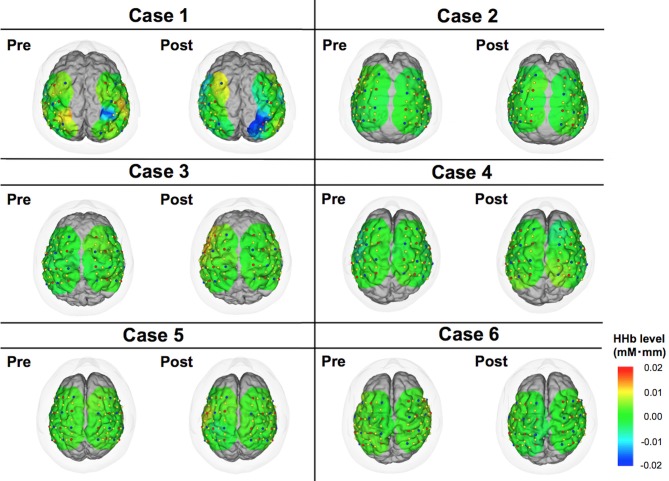
**Color maps of deoxy-hemoglobin (HHb) levels comparing pre- vs. post-DBS states.** These images indicate cortical activities at 15 s from the start of motor task. Relative changes compared to the status at 0 s are demonstrated in this figure.

For individual analyses, we used the software pre-loaded on the NIRS machine. Optode location was superimposed on the three-dimensional brain image from the volumetric T1-weighted image used for surgical planning. Spatial registration was performed using 15 pictures covering all probes and points of references, including the nasion and bilateral tragi (Figure 1). Subsequently, changes in HbO and HHb levels were color-mapped onto each brain image. Prior to the analysis, baseline correction was performed for individual analysis by averaging the starting point data of seven blocks and correcting them to zero. A temporal low-pass cut-off frequency was set at 0.1 Hz to remove high-frequency noise from fNIRS detectors associated with systemic oscillations from cardiac signal and respiration. The relative changes in the hemoglobin signal were denoted in arbitrary units of mM × mm. The color maps of our cohort were shown in Figures 2, 3, 4.

A group analysis of fNIRS data was performed using the NIRS-SPM software (KAIST, Korea) operated in the MATLAB environment (Mathworks, Natick, MA, USA; Ye et al., 2009). This software enables an analysis similar to the statistical parametric mapping of fMRI. In the analyzing process, signal distortion due to breathing or movement of the subject was first corrected by hemodynamic response function and the wavelet minimum description length detrending algorithm incorporated in the NIRS-SPM software. A predicted model of HbO and HHb response was then generated based on the general linear model using data from the experimental condition. Using the same spatial information as that in the spatial registration for the individual analysis, the NIRS-SPM aligned the mean optode positions of six participants according to the Montreal Neurological Institute’s standardized brain coordinate system (Figure 1F). The activation map based on the Hemoglobin level changes was then computed for the standardized brain. SPM *t*-statistic maps were produced, and HbO levels were considered significant at the uncorrected threshold of *p* < 0.01. Left/right information was flipped in the right-sided DBS cases for group analysis.

### Statistical Analysis of Clinical Outcomes

We performed a Wilcoxon signed rank test to compare the pre- and post-DBS UPDRS off medication scores. For the statistical analysis, we used SPSS version 21.0 (IBM Corp., Armonk, NY, USA).

## Results

### Clinical Outcomes

Preoperative motor evaluation showed 58.8 ± 11.1% improvement on the levodopa challenge test, as total scores on the UPDRS motor scale were 48.5 ± 11.1 and 19.7 ± 5.6 in the off- and on-medication states, respectively. Postoperatively, total UPDRS motor scale scores had improved to 26.8 ± 16.6 (*p* < 0.05) and 22.2 ± 8.6 (*p* < 0.05) at 1- and 6-month follow-up, respectively. These clinical outcomes and DBS programs at the point of the fNIRS experiment are summarized in Table 2. As for adverse events, only one patient (case 5) had wound dehiscence and underwent debridement. Otherwise, no serious adverse events were reported.

**Table 2:** **Clinical outcomes**.

	Baseline			1 month	6 months	DBS programming at 1 month evaluation			
Case	Baseline off-medication	Baseline on-medication	% improvement	Off-medication/on-DBS	Off-medication/on-DBS	Active contacts	Voltage	Pulse width	Frequency
1	36 (1)	21	41.7	26 (0)	13	C+/1-	2.8	60	180
2	39 (1)	18	53.8	31 (0)	24	C+/1-, 3-	2.0	60	125
3	55 (2)	17	69.1	5 (1)	18	C+/1-	2.3	60	130
4	65 (3)	29	55.4	51 (0)	33	C+/1-	3.2	60	180
5	53 (2)	21	60.4	12 (1)	14	C+/2-	2.0	60	130
6	43 (2)	12	72.1	36 (3)	31	C+/1-	1.5	60	130
**Mean ± SD**	48.5 ± 11.1	19.7 ± 5.6	58.8 ± 11.1	*26.8 ± 16.6	*22.2 ± 8.6				

The numbers in the parentheses at baseline and 1 month are item 24 (hand movement subscore) of the UPDRS on the treated side of the body. The subscore ranges 0–4 (0-Normal; 1-Mild slowing and/or reduction in amplitude; 2-Moderately impaired. May have occasional arrests in movement; 3-Severely impaired. Frequent hesitation in initiating movements or arrests in ongoing movement; 4-Can barely perform task). “C” represents “case” for monopolar stimulation. *These postoperative scores were significantly improved compared to baseline off-medication score. A Wilcoxon signed rank test was performed for the statistical analysis.

### Representative Case (Patient 3)

Patient 3 was a woman of 65 years of age when she underwent left unilateral GPI DBS. Her baseline UPDRS motor scores were 17 and 55 in the on- and off-medication states, respectively. Preoperatively, her main problems were festination in the off-medication state, on/off motor fluctuation, and leg tremor worst on the right side. Her motor symptoms dramatically improved following DBS surgery, and her postoperative UPDRS motor scores were 5 and 18 with stimulation in the off-medication state at 1- and 6-month follow-up sessions, respectively. In the fNIRS experiment, motor performance in this case was changed from 2 (moderately impaired) to 1 (mildly impaired) as shown in the UPDRS subscore (item 24). NIRS showed that her HbO levels were higher in the operated-upon left hemisphere, postoperative changes seemed to occur in tandem with the improvement of motor function (Figure 2).

### Individual Analysis

The motor task performance was scored by UPDRS item 24 (hand movement score). The task performance score ranges from 0 to 4 with four being the worst symptom, and the results are summarized in Table 2. The task performance was improved in all cases except for case 6. Based on the HbO levels, there seemed to be a tendency that the cortical activity in the operated hemisphere was more activated compared to pre-operative state (Figure 3). On the other hand, color maps of HHb levels failed to show as dramatic changes as those of HbO levels (Figure 4). Even though the motor tasks were different, changes in the HHb levels in our experiment seemed to be consistent with previous studies using a similar NIRS system that have not shown significant changes in HHb levels in association with the motor task (Mihara et al., 2012; Fujimoto et al., 2014).

### Group Analysis

**Figure 5:**
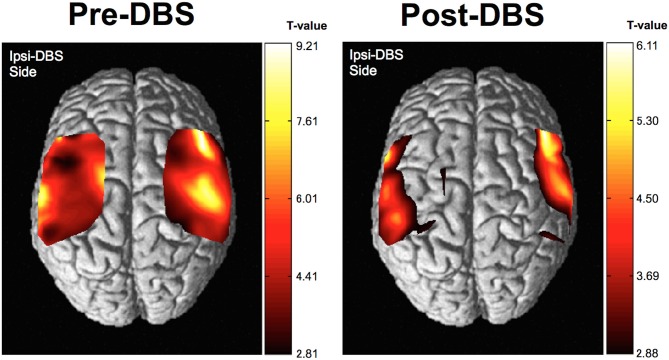
**Results of near infrared spectroscopy (NIRS)-SPM group analysis.** Cortical activity, as determined by NIRS-SPM, was relatively increased in the primary motor area in the operated-upon hemisphere postoperatively. Higher *t*-values represent relatively higher cortical activity than areas with lower *t*-values in this figure.

The group analysis using NIRS-SPM showed that, cortical activity based on the HbO level changes was shown to be of almost equal intensity throughout the investigated areas prior to DBS surgery. However, the cortical activity associated with movement of the hand was more focused in the corresponding motor area following surgery. The motor-related activity was more focused in the motor cortex, postoperatively (Figure 5). *T*-statistics of HHb level changes failed to show significant voxel on the map.

## Discussion

We investigated the cortical activity changes in both hemispheres of patients with PD that were received unilateral therapeutic GPI DBS, based on a 6-month follow-up of clinical outcomes. Previous studies have shown that remote areas with functional connectivity to the subthalamic nucleus or GPI could be modulated using electrical stimulation (Hashimoto et al., 2003; de Hemptinne et al., 2015). Recent neurophysiological studies have shown that there is an abnormal synchronization of the beta-band oscillation between the subthalamic nucleus and the motor cortex in PD (de Hemptinne et al., 2015; Kondylis et al., 2016), and that DBS may modulate the global network of the brain. Most of these studies have investigated patients with PD who received bilateral DBS of the subthalamic nucleus. Our data also suggests that DBS may modulate the global networks involved in motor activities. As a novelty of this study, we believe that investigated the effects of unilateral DBS, and this may be preferable as a means of revealing the influence of each DBS site on the global network.

The combined use of NIRS and DBS has been investigated in only one other study. However, while this previous study by Sakatani et al. (1999) showed increased levels in the prefrontal areas with increased electrical intensity of DBS from 0 to 10 volts in subjects with PD, merely increasing stimulation intensity is no longer considered to be of therapeutic benefit. Additionally, this previous study used a single-channel NIRS system. Our study aimed to exceed these standards by detecting task-related cortical activities with therapeutic DBS conditions using the multi-channel NIRS system, yielding clinically-relevant imaging results of superior spatial resolution.

Previous task-related fMRI studies showed decreased activity in the supplementary motor area and relative hyperactivity in the primary sensorimotor and premotor areas of subjects with PD compared to healthy controls (Sabatini et al., 2000; Haslinger et al., 2001). Hypoactivation of the sensorimotor area was considered to be related to the festination to initiate voluntary movements. Our data show a relative increase in the activity of the primary motor areas of the ipsi-DBS side with clinical improvement as shown by the changes in HbO levels. Moreover, our results are consistent with previous fMRI studies.

One noteworthy advantage of fNIRS in clinical use is its ability to provide patients with real-time visual feedback of cortical activity, although this feature was not used in the present study. Several studies have investigated the potential of a real-time neurofeedback system for neurorehabilitation (Holper and Wolf, 2010; Fazli et al., 2012; Mihara et al., 2013). The real-time visualization of cortical activity may be useful for intraoperative monitoring. Recent studies emphasize the benefits of a closed-loop DBS system, but the best biomarker to detect changes in brain activity remains to be determined (Johnson et al., 2016; Rossi et al., 2016). However, a multi-channel NIRS system might spur the development of a new generation of DBS systems. Our data demonstrated the potential of fNIRS to detect cortical activity, as we hypothesized. Further studies are warranted to develop the system for real-time neurofeedback during DBS surgery.

In the present study, we showed the potential of the NIRS system for investigating patients with PD who have undergone DBS surgery. Nonetheless, there are important limitations of our study. First, current NIRS systems are unable to investigate the activity of subcortical areas such as the basal ganglia. Second, several studies have investigated the effects of a microlesion following DBS surgery (Mann et al., 2009; Morishita et al., 2010; Okun et al., 2012), and a few studies determined that the microlesion effect may postoperatively continue for up to 6 months (Mann et al., 2009; Morishita et al., 2010). The NIRS data reported here was obtained at a 1-month follow-up, thus we cannot exclude the possibility that the NIRS findings that we observed may represent changes in cortical activity that are attributable to the microlesion effect. Third, it should be noted that our NIRS findings may represent an improvement in the produced movement itself, and this question should be addressed in a future study. Such an improvement would indicate that the fNIRS system allows for a more objective evaluation of the clinical effect of therapeutic DBS compared to other clinical scales. Fourth, we did not perform sophisticated statistical analysis to address false discovery rate, Bonferonni correction and controlling type I error in this pilot study (Tak and Ye, 2014). Finally, as only six PD cases were included in our study, and we have not related the performance status with the fNIRS data. Further studies with larger samples and more sophisticated study design are warranted to confirm the reproducibility of our experiment.

## Conclusion

To our knowledge, our study is the first to use a multi-channel NIRS system to measure the changes in cortical activity following unilateral GPI DBS. In this pilot study, we show the changes in the motor-associated cortical activity following DBS surgery. Moreover, we conclude that therapeutic DBS promoted the remodeling of neuronal networks and changes in cortical activity in association with symptomatic improvements.

## Author Contributions

TM contributed to conception of the study and data collections, and wrote the manuscript. MH collected the data, and reviewed the manuscript. KS collected and analyzed the data. YT reviewed the manuscript. HA reviewed the manuscript. TI supervised the study and reviewed the manuscript.

## Conflict of Interest Statement

The authors declare that the research was conducted in the absence of any commercial or financial relationships that could be construed as a potential conflict of interest.

## Abbreviations

DBS: deep brain stimulation
fNIRS: functional near infrared spectroscopy
fMRI: functional magnetic resonance imaging
GPI: globus pallidus interna
HbO: oxy-hemoglobin
HHb: doxy-hemoglobin
MRI: magnetic resonance imaging
NIRS: near infrared spectroscopy
PD: Parkinson’s disease
UPDRS: Unified Parkinson’s Disease Rating Scale.
